# When Blood Remembers Its Sex: Toward Truly Personalized Transfusion Medicine

**DOI:** 10.3390/jpm15120592

**Published:** 2025-12-03

**Authors:** Sotirios P. Fortis, Styliani Kokoris, Pavlos Kelepousidis, Georgios Dryllis, Maria-Aspasia Kosma, Theodoros Pittaras, Anastasios G. Kriebardis, Serena Valsami

**Affiliations:** 1 Laboratory of Reliability and Quality Control in Laboratory Hematology (HemQcR), Department of Bio-Medical Sciences, School of Health & Caring Sciences, University of West Attica (UniWA), 12243 Athens, Greece; sfortis@uniwa.gr (S.P.F.); gdryllis@uniwa.gr (G.D.); mkosma@uniwa.gr (M.-A.K.); akrieb@uniwa.gr (A.G.K.); 2 Laboratory of Hematology and Blood Bank Unit, “Attikon” University General Hospital, School of Medicine, National and Kapodistrian University of Athens, 11521 Athens, Greece; skokori@med.uoa.gr; 3 Postgraduate Program “Thrombosis, Bleeding, and Transfusion Medicine”, School of Medicine, National and Kapodistrian University of Athens, 11521 Athens, Greece; pavloskelep@gmail.com; 4 Hematology Laboratory and Βlood Bank, Aretaieion Hospital, National and Kapodistrian University of Athens, 11528 Athens, Greece; tpittaras@med.uoa.gr

**Keywords:** blood transfusion, donor, personalized medicine, gender, sex, blood products

## Abstract

**Background**: Biological sex differences are well-recognized as non-negligible factors in implementing precision medicine practice. Sex chromosomes influence protein expression and signaling, and thus cellular pathways are often regulated differently. Additionally, the importance of sex as a biological variable has gained significant traction in biomedical research, including transfusion medicine. Regarding transfusion medicine, several studies reveal the role of gender in blood transfusion, blood donors’ behavior towards donation, blood products’ composition and storage, transfusion therapy, and possibly post-transfusion patient outcomes. **Methods**: In this review, the role of sex and gender in the whole transfusion chain (from the blood donor to the blood product and the patient) is assessed and summarized using data from observational studies, registry analyses, and randomized trials. **Results**: Female donors face higher deferral rates due to biological factors (iron deficiency, low hemoglobin, pregnancy) and sociocultural factors (caregiving responsibilities, misinformation). However, women are more likely to donate based on empathy, moral duty, or community responsibility and are more consistent in sustaining voluntary donation during crises. Men donate more frequently, typically driven by external motivators, and provide red blood cell (RBC) products with higher hemoglobin content, whereas RBCs from female donors exhibit greater metabolic stability and reduced hemolysis during storage. Plasma from multiparous women possibly contains alloantibodies associated with adverse transfusion reactions, namely transfusion-related acute lung injury (TRALI). Platelet function also varies by sex, though its possible clinical impact is still unknown. Although observational studies suggest sex-mismatched transfusions are associated with increased morbidity and mortality—particularly in transfusions from female donors to male recipients—large registries and randomized controlled trials show inconsistent or negligible effect on survival. **Conclusions**: Donor and recipient sex are emerging variables of possible clinical importance in transfusion practice. Incorporating sex-informed insights into donor recruitment, blood product handling and transfusion policies may improve safety while advancing precision medicine. Further large-scale trials are needed to elucidate the impact of sex in transfusion, identify and eliminate possible risks, and bridge the gap between biological insights and clinical practice.

## 1. Introduction

The era of personalized precision medicine has become a cornerstone of modern healthcare, aiming to tailor medical decisions and therapeutic strategies to individuals’ unique biological, genetic, and environmental characteristics [1,2]. Although early approaches focused mainly on genome-wide profiling and molecular diagnostics [3], increasing evidence demonstrates sex and gender as pivotal factors determining health outcomes [4,5]. Biological, hormonal, and immunological differences between men and women modulate susceptibility to disease, response to treatment, and the safety and efficacy of therapy [6,7]. Accordingly, sex-based variation also extends into the field of transfusion medicine [8,9].

Blood transfusion is essential to modern clinical practice for patients needing surgery, trauma care, cancer treatment, and other life-saving procedures that would otherwise be impossible [10,11]. Blood transfusion depends on blood donation, and according to the World Health Organization (WHO), “Safe and adequate global blood supply can only be achieved through voluntary and unpaid blood donation” [12]. Although blood safety and donor recruitment strategies have improved substantially over recent decades, maintaining an adequate blood supply remains a major challenge, especially in low- and middle-income countries [13,14,15,16]. Donated blood is processed into various products, mainly red blood cells, platelets, and plasma for transfusion for different clinical uses.

Increasing evidence highlights the influence of sex and gender across the entire transfusion chain. Sex refers to biological attributes, whereas gender denotes socially constructed roles and behaviors [17]. Studies have reported sex-related differences in donor behavior towards donation and motivation, eligibility criteria for blood donation, the composition and storage characteristics of blood products, and the susceptibility of specific products to transfusion-related complications [18,19,20]. Furthermore, biological, hormonal, and immunological factors that differ between sexes affect blood product composition and recipient immune responses and may be important but yet under-explored determinants of patient outcomes [6]. This review summarizes the current evidence and discusses knowledge gaps concerning the role of sex and gender in transfusion medicine, spanning the continuum from donor to recipient.

## 2. The Role of Gender in Donor Motivation and Behavioral Dynamics

Blood donation behavior is not a static feature and evolves through time, influenced by the psychology of the individual, personal experiences, sociocultural context, and spontaneous events. Gender-based behavioral differences further refine motivational patterns in blood donation. In many high-income countries, gender distribution among donors is relatively balanced—for example, women comprise 50–53% of donors in France and the UK, while in various parts of Asia, the proportion of women is lower among donors [21,22]. In Greece, in particular, women represent less than one-third of total donors. Research suggests that female donors are more likely to internalize prosocial values and donate based on empathy, moral duty, or community responsibility, as many studies indicate that altruism—helping others without expectation of reward—remains a key driver of behavior [23].

Conversely, male donors are often more responsive to external motivators such as perceived health benefits, societal expectations, and emergency appeals [24,25]. These tendencies are consistent with the Theory of Planned Behavior, which explains blood donation intention through attitudes, social norms, and perceived behavioral control—elements that manifest differently across genders [26].

On the other hand, barriers to donation include a lack of awareness regarding the eligibility criteria, poor access to donation centers, and health-related data (e.g., anemia, low body mass), and most of these barriers are prevalent among women. [25,27,28]. Misconceptions surrounding menstruation, physical strength, or the safety of donation further limit participation, while social roles related to family and reproduction impose additional constraints [29,30].

Importantly, donation behavior is sensitive to the larger societal context, and gender differences play a critical role in shaping donor participation. Large surveys demonstrate that women are more likely to report altruistic and socially driven motives for donating blood, whereas men more often cite pragmatic or health-related reasons [31,32,33]. Despite this, women frequently face structural barriers, including higher deferral rates due to low hemoglobin, pregnancy, or lactation, which help explain their lower overall representation among repeat donors in many regions [27,34]. Donation systems face dual pressure during crises—rising demand and reduced supply—such as during pandemics, natural disasters, and mass casualty events. For instance, the COVID-19 pandemic caused a significant drop in donor turnout due to lockdowns, fear of infection, and reduced mobility [10,14]. Our group has further shown that during the Greek economic crisis (2015–2016), men comprised the majority of donors (68.2%), yet their donation frequency declined more sharply, while women, though fewer, remained more consistent in sustaining voluntary donation [35]. In contrast, during the COVID-19 pandemic (2020–2021), women represented the majority of donors (62.7%) and were more likely than men to maintain active participation in blood donation, along with younger individuals and students [36]. This reversal reflects a broader global trend: WHO estimates indicate that only one-third of blood donations worldwide come from women, though proportions vary substantially across regions [37]. In some countries, such as the United States, women have recently become the majority of donors (54.1% in 2021) [38]. Taken together, these findings suggest that although women may be underrepresented in stable times due to higher deferral rates, their engagement is resilient and can become a leading force in sustaining the blood supply during crises.

## 3. The Role of Donor Sex in Blood Donor Eligibility Criteria

Blood donation eligibility is governed by strict criteria to ensure the safety of both donors and recipients. Exclusion or deferral from donation can occur due to biological, medical, behavioral, or situational factors, with notable differences between men and women influencing these outcomes.

Women face specific biological barriers to donation. They are at increased risk of iron deficiency due to menstruation and lower iron stores and have lower normal hemoglobin levels. Thus, the threshold for blood donation is also lower (≥12.5 g/dL) compared to men (≥13.5 g/dL) [39,40]. However, this often leads to temporary deferral, particularly as their total blood volume is lower, affecting donation ability and recovery [41]. Common reasons for deferral among female donors besides low hemoglobin levels—frequently tied to iron deficiency or pregnancy-related anemia—include hypotension and low body weight. Studies show that frequent causes of deferral, such as vasovagal reactions and low hemoglobin levels, are significantly more common in women weighing under 68 kg [25,42].

Female donors also have a greater risk of producing anti-HLA antibodies (particularly from pregnancy), which can increase the risk of transfusion-related adverse events, including TRALI [43]. This has led many blood banks worldwide to prefer male donors for plasma and platelet donations to reduce the risk of TRALI in recipients. Post-Transfusion Purpura (PTP), a rare immune-mediated complication, also disproportionately affects multiparous women due to alloimmunization during pregnancy, mediated by antibodies against platelet antigens (e.g., anti-HPA-1a), further justifying exclusion in specific cases [44].

Men donate blood more frequently than women due to higher levels of hemoglobin and lower incidences of iron deficiency [45,46]. Additional exclusion criteria apply universally. Donors must meet minimum weight requirements (usually ≥50 kg) to ensure safe blood volume loss, with lower weights more commonly deferring women [25].

Targeted interventions can mitigate some deferrals. For women, increasing donation intervals to over 19 weeks, monitoring ferritin every three donations annually, iron supplementation, pre-donation hydration, and isometric exercises have been proposed to reduce deferrals [25,42]. Low ferritin levels, even with acceptable hemoglobin, are increasingly monitored to prevent donor iron depletion, particularly in frequent female donors [25]. Understanding these diverse reasons—biological, sex-specific, medical, and behavioral—is essential for optimizing donor selection and maintaining a safe blood supply.

## 4. Sex-Related Differences in Blood Products: Focus on RBCs, Platelets and Plasma

**Figure 1:**
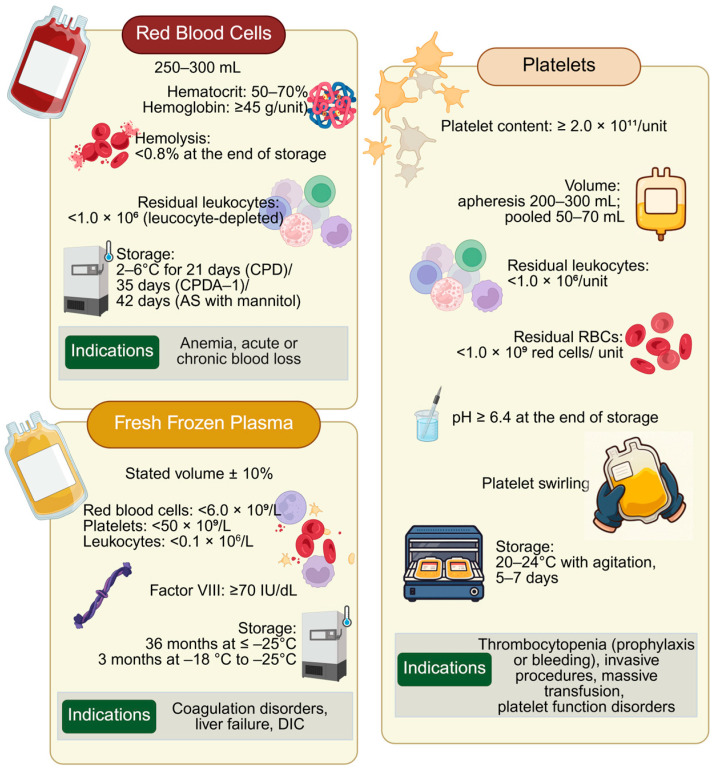
Indications and storage requirements of major blood products. Quality control (QC) parameters, storage conditions, and principal clinical indications for the main blood components: Red Blood Cells (RBCs), Fresh Frozen Plasma (FFP), and Platelets (PLTs). Adapted from the Council of Europe, Guide to the Preparation, Use and Quality Assurance of Blood Components, 21st Edition (2023) [47]. Created in BioRender. Fortis, S. (2025) https://BioRender.com/mttdkm8 (accessed on 30 October 2025). Abbreviations: RBCs, red blood cells; DIC, disseminated intravascular coagulation; vWD, CPD, citrate–phosphate–dextrose; CPDA-1, citrate–phosphate–dextrose–adenine solution; AS, additive solution.

Blood products are essential for a wide range of clinical indications across diverse clinical settings, and the number of available products for transfusion continues to increase. This review focuses on the three main blood products: red blood cells (RBCs), platelets (PLTs), and fresh frozen plasma (FFP). Figure 1 summarizes blood product requirements, quality control (QC) parameters, storage conditions, and principal clinical indications of major blood products, based on the Guide to the Preparation, Use and Quality Assurance of Blood Components, 21st Edition [47].

### 4.1. RBCs Blood Products for Transfusion—Sex-Related Differences

Donor sex has emerged as a key biological variable influencing the storage quality, composition, and function of blood products—including RBCs, FFP, and platelets—by imparting distinct molecular and physiological traits that shape their overall composition and performance. In RBC products, hemoglobin concentration is highly sex-dependent: 13.8–17.2 g/dL in males versus 12.1–15.1 g/dL in females (driven by androgen-mediated erythropoiesis in males versus estrogen-modulated iron homeostasis in females) [39,48,49]. Primary processing focuses on a common standard of 50–60 g hemoglobin per RBC unit such that female donor units may be 5–10% lower in hemoglobin, thereby possibly decreasing their ability to deliver oxygen [50]. The hematocrit, Hb level, mean corpuscular volume (MCV), and transferrin levels are lower in females compared to males [51]. The work of Angelo D’Alessandro further unfolds sex-specific metabolic profiles in stored RBCs: he showed that RBCs from female donors demonstrate higher glycolytic flux and lower levels of oxidative stress markers (including reduced glutathione depletion) when compared to those from male donors. On storage day 42, female RBCs also possess greater membrane integrity (i.e., less hemolysis), which may be attributed to differences in lipid peroxidation [18,52]. In addition to lipid peroxidation differences, hormonal influences may also contribute to the enhanced membrane resilience of female RBCs [53]. Progesterone has been suggested to exert a protective effect on the red cell membrane by inhibiting calcium influx, while estradiol may enhance antioxidant capacity [54,55]. Furthermore, higher sphingomyelin content in erythrocyte membranes has been associated with improved membrane stability and reduced fragility [56,57]. Although women’s RBC features contribute to enhanced storage stability and possibly better post-transfusion performance according to in vitro studies [58,59], these changes rarely impact clinical recovery rates, which are around 75–85% for both sexes [60]. Additionally, women’s RBCs generally are larger in size and contain higher intracellular levels of reactive oxygen species (ROS) and calcium, along with lower mean corpuscular hemoglobin concentration (MCHC) and reduced membrane stiffness compared to male RBCs [61].

A substantial and expanding body of research has documented the structural and functional damage that RBCs undergo during hypothermic storage, commonly referred to as the “storage lesion” [62,63,64]. More recently, attention has shifted toward the role of donor-related factors in influencing storage outcomes. Several studies analyzing quality control data from over 16,000 male and 11,000 female donors demonstrated that donor sex and age significantly affect hemolysis levels at the end of 42 days of storage. RBCs from female donors are less susceptible to storage, osmotic and mechanical hemolysis compared to those from males, while male RBCs exhibit reduced deformability, possibly related to testosterone exposure, which has been shown to enhance hemolytic susceptibility [65].

**Figure 2:**
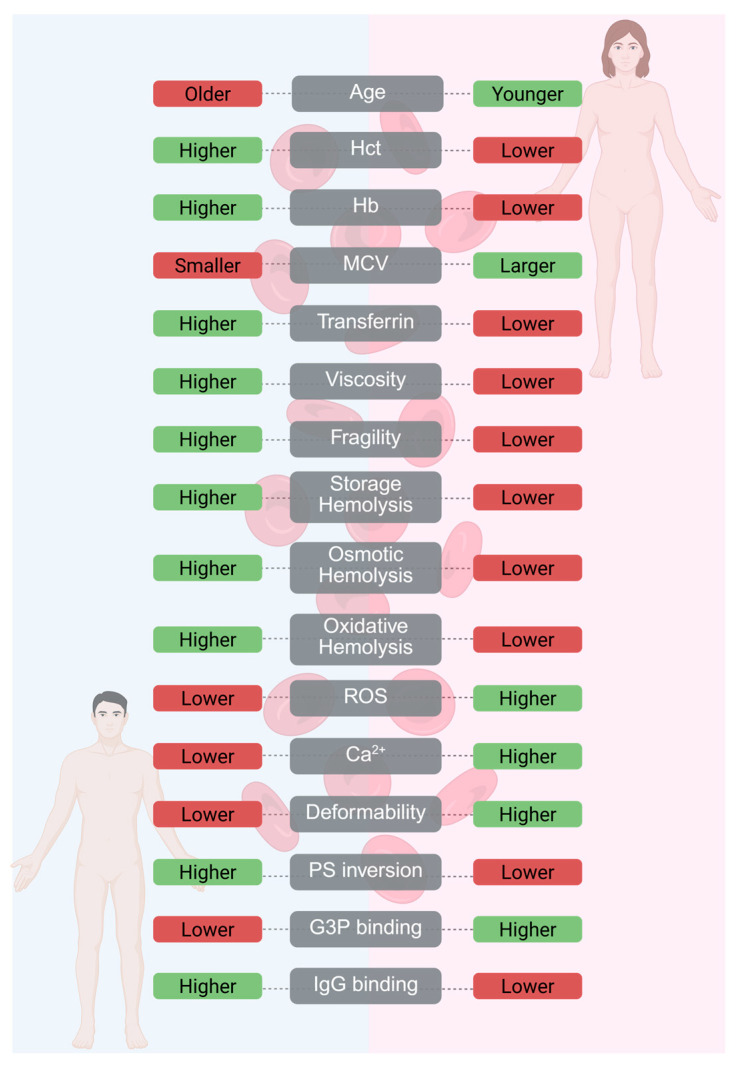
Parameters Highlighting Sex Differences in Red Blood Cell Structure and Function. This figure illustrates sex-based differences in red blood cell (RBC) characteristics between males and females. Parameters include hematological values, markers of RBC aging, hemolysis susceptibility (storage, osmotic, oxidative), intracellular stress indicators (ROS, Ca^2+^), and membrane properties. Male RBCs tend to be older, smaller, and more fragile, with higher hemolysis and IgG binding. Female RBCs are generally younger, larger, more deformable, and show higher G3P binding and stress resilience. Adapted from [73]. Created in BioRender. Fortis, S. (2025): https://BioRender.com/mttdkm8 (accessed on 30 October 2025). Abbreviations: MCV: Mean Corpuscular Volume; Hct: Hematocrit; Hb: Hemoglobin; ROS: Reactive oxygen species; Ca: Calcium; PS: Phosphatidylserine; G3P: Glyceraldehyde-3-Phosphate; IgG: Immunoglobulin G.

Besides hormonal and metabolic effects, the interindividual variation in RBC storage quality also depends on genetic determinants. A recent large genome-wide association study (GWAS) of more than 12,000 blood donors (REDS-III RBC-Omics) identified 27 loci strongly associated with oxidative, osmotic, and cold-stress-induced hemolysis, including strong associations with variants in G6PD, GLRX, and GPX4 [66]. Additional studies demonstrate that G6PD deficiency predisposes RBCs to oxidative stress and increased hemolysis during storage, compromising metabolic stability and post-transfusion recovery [67,68,69,70]. These findings support that genetically determined differences in red cell metabolism and antioxidant capacity interact with donor-sex effects, thus contributing to the explanation of the heterogeneity observed in RBC storage behavior. The age of RBCs in storage units is closely associated with donor sex, as women generally have a higher proportion of younger circulating red blood cells [71]. This age profile may be influenced by menstrual blood loss, which stimulates erythropoiesis and introduces a fresher population of RBCs into circulation [49]. The presence of fewer aging-related markers in female RBCs further supports the idea that women generally have a younger circulating RBC pool than men [51,61]. IgG binding to RBCs differs between sexes, with studies showing that male RBCs typically exhibit higher levels of surface-bound IgG than female RBCs, possibly due to greater RBC aging or membrane remodeling [72,73,74]. Female RBCs show greater binding of glyceraldehyde-3-phosphate (G3P), which may enhance metabolic stability during storage and post-transfusion circulation [18,73,75]. Blood viscosity, influenced by RBC properties, is also higher in males compared to females, which could impact flow dynamics and oxygen delivery in recipients [76]. However, it is worth noticing that independent of RBC age, in clinical transfusion practice, blood from male donors, due to the fact that it has a significantly greater hemoglobin concentration and hematocrit compared to blood from female donors [73,77,78], leads to greater hemoglobin increases in recipients after transfusion [73,79]. Sex-related RBC differences are illustrated schematically in Figure 2.

### 4.2. Plasma Products for Transfusion, Sex-Related Differences

The acellular component, plasma, also exhibits several prominent sex-specific differences. Female donors with prior pregnancies develop anti-HLA and anti-HNA antibodies in 10–25% of cases, representing alloimmunization resulting from fetomaternal hemorrhage during pregnancy [80]. Notably, the extent of alloantibody formation appears to follow a dose–response relationship with the number of pregnancies. Multi-parity, stillbirths, and miscarriages are associated with progressively higher anti-HLA antibody levels in female donors [80,81]. Absent from the majority of male plasma, these antibodies increase the risk of TRALI by inducing neutrophil activation in patients after transfusion. Consequently, transfusion policies often promote the use of male or nulliparous female plasma to reduce transfusion-related respiratory complications [81]. It is worth noticing that proteomic analyses reveal additional differences: plasma from females contains higher levels of acute-phase proteins (e.g., haptoglobin), whereas the levels of coagulation factors (e.g., factor VIII) are reduced compared to male plasma, reflecting the influence of estrogens and testosterone, respectively [82,83,84].

### 4.3. Platelet Products for Transfusion, Sex-Related Differences

Platelets in humans exhibit sex differences in proteins, mRNAs, and miRNAs, supporting the concept that sex-related variations in platelet reactivity are, at least in part, intrinsic to platelets themselves [85,86]. Platelets from female donors display higher baseline activation (i.e., higher P-selectin levels of platelets) due to estrogen-induced upregulation of integrin signaling [87]. According to Hadley et al. (2022), platelet metabolism varies with donor sex and age. Platelets from older males exhibited higher ATP levels and purine breakdown products, as well as elevated arachidonic acid and other fatty acids, indicating sex-linked metabolic differences that may influence platelet function [88]. However, the clinical relevance of these differences remains to be elucidated.

## 5. Impact of Donor–Recipient Sex Matching on Transfusion Outcomes

### Donor–Recipient Sex Matching in RBC Transfusions

The potential association between sex-mismatched red blood cell (RBC) transfusions and adverse clinical outcomes has been explored in several studies [19,89,90,91,92,93,94,95,96,97,98,99]. Recent observational studies suggest that any detrimental effects may be confined to the immediate post-transfusion period [89,90,91,92,93], whereas randomized trials have questioned their clinical significance [19]. Overall, the evidence remains heterogeneous: while some studies report increased risk, others report no significant association after adjustment for confounding factors.

In an early Dutch cohort of 31,118 recipients [89], male recipients of female donor blood (F→M)—particularly those aged ≤55 years—had higher mortality (HR ~1.8), whereas female recipients of male donor blood (M→F) showed no clear harm. Using the same registry, Caram-Deelder et al. reported that excess risk in men was confined to ever-pregnant female donors (F(parous)→M; HR ~1.13 in the single-donor-type cohort; ~1.08 per unit in the full cohort), with no effect from nulliparous women [92]. In Sweden, Bjursten et al. studied 9907 cardiac surgery patients and found increased mortality with F→M transfusions, particularly when RBC units were not leukoreduced [95]. By contrast, Desmarets et al. observed worse unadjusted survival in 2715 French cardiac surgery patients given sex-mismatched RBC units (HR ~2.28), especially M→F, but this effect disappeared after adjustment for confounding factors [94].

A systematic review pooling five observational datasets (86,737 patients) found a modest mortality increase with sex-mismatched RBC transfusions (HR ≈ 1.13), but the certainty of evidence was very low, and specific donor–recipient pairings were not distinguished [93]. Mechanistic ICU studies offer some biological plausibility: Alshalani et al. found higher syndecan-1 and thrombomodulin after sex-mismatched transfusion in 69 critically ill patients, suggesting endothelial glycocalyx injury [98]. In a larger cohort of 6992 ICU patients (403 receiving single-sex transfusions), F→M transfusions were associated with increased ICU mortality (OR 2.43) [96]. Similar trends have been reported for acute respiratory distress syndrome (ARDS), although statistical significance was not consistently reached [96]. Recently, Li et al. studied 500 critically ill patients and found no donor-sex effect on sepsis, though female recipients had greater hemoglobin increments when transfused with male donor blood [97]. A registry-based causal inference analysis likewise suggested a modest survival advantage for sex-matched transfusions, although this finding may reflect residual confounding and did not distinguish between specific donor–recipient pairings [99]. An ongoing trial, SexMATTERS (NCT06840756), will directly test the effect of sex matching in critically ill patients.

Population-based studies provide mixed results. In Canada, a cohort of 30,503 patients receiving 187,960 transfusions from 80,755 donors showed that female donor blood was associated with an 8% higher mortality risk compared with male donor blood (adjusted HR 1.08, 95% CI 1.06–1.09; *p* < 0.001) [90]. In contrast, much larger Scandinavian cohorts—including 968,264 patients [91] and 368,778 patients [50]—found no association between donor sex or parity and survival. In the former, even among those receiving multiple female donor units, crude analyses showed no effect, and any apparent differences disappeared after adjustment for transfusion volume. The latter study likewise found no link with 2-year mortality and suggested that lower hemoglobin content in female donor RBCs, rather than donor sex per se, may have confounded earlier findings. Finally, the multicenter randomized trial by Chassé (2023), involving 8719 patients randomly assigned to receive RBC units from either male donors or female donors, showed no significant difference in survival between a transfusion strategy involving RBC units from female donors and a strategy involving RBC units from male donors (HR 0.98) [19]. A summary of the key findings from the aforementioned studies is provided in Table 1.Taken together, observational data point to a potential risk for male recipients of female donor blood, particularly from parous women, but the largest adjusted cohorts and the only randomized trial to date suggest that donor–recipient sex mismatch does not have a meaningful impact on survival in routine transfusion practice.

## 6. Conclusions

In the era of personalized transfusion medicine, accumulating evidence highlights the influence of sex and gender on various aspects of transfusion medicine, including donor behavior, blood product composition and storage, transfusion therapy, and potentially post-transfusion outcomes. Published data suggest that donor-sex-related factors (i.e., variations in hematological parameters, immune responses, and hormonal levels) affect the composition and storage characteristics of blood products, whereas the sex of the recipient may modulate immune responses post-transfusion. However, evidence regarding the clinical relevance of these differences remains inconsistent. In summary, it is well established that donor sex influences the characteristics of blood products. Specifically, RBC products from male donors tend to have higher hemoglobin content [79], whereas female RBCs are more resistant to hemolysis and oxidative damage during storage [18,20]. These properties may confer advantages in patients requiring multiple or long-term transfusions, such as neonates or individuals with hematologic disorders. Regarding plasma-containing blood products, female donor plasma—particularly from multiparous women—often contains antibodies such as anti-HLA and anti-HNA, which can trigger severe transfusion reactions, most notably TRALI. Implementation of male-only plasma policies has markedly reduced the incidence of TRALI, as evidenced by large international hemovigilance datasets [100,101]. Nevertheless, despite robust evidence supporting this preventive measure, several countries still lack explicit national guidelines on donor sex selection for plasma management, underscoring the need for harmonized international protocols [81]. Moreover, although observational data demonstrated that transfusion of sex-mismatched blood—especially from female donors to male recipients—might be linked to adverse outcomes such as increased mortality and endothelial activation [90,96], randomized controlled trials have not consistently confirmed these associations [19,91]. Notwithstanding these inconsistencies, the potential for clinically relevant effects in specific subgroups (e.g., younger males or critically ill patients) highlights the importance of continued vigilance and systematic hemovigilance reporting to identify high-risk populations who might benefit from sex-tailored transfusion strategies.

In conclusion, accumulating evidence underscores that both donor and recipient sex represent emerging determinants of possible clinical relevance in transfusion medicine. Integrating sex as a biological variable into donor recruitment, blood product processing, and transfusion policy development may enhance transfusion safety and promote the principles of precision medicine. Future large-scale, well-designed studies are essential to elucidate the impact of sex differences in transfusion, identify and eliminate possible risks, and thereby close the translational gap between biological insight and clinical practice.

## Figures and Tables

**Table 1:** Summary of studies evaluating the impact of donor–recipient sex mismatch in red blood cell (RBC) transfusion on clinical outcomes.

Study	Design/Population (*n*)	Male Recipient from Female Donor (F→M)	Female Recipient from Male Donor (M→F)	General Sex-Mismatched (if no Detail)
Middelburg 2011 [89]	Observational, NL, 31,118 recipients	Increased mortality in men ≤55 years (HR ~1.8)	No significant effect	HR ~1.2 overall (not significant)
Caram-Deelder 2017 [92]	Observational, NL, 31,118 recipients	Increased mortality if donor parous female (HR ~1.13 single-donor; HR ~1.08 per unit, full cohort)	No effect	–
Bjursten 2016 [95]	Cardiac surgery, SE, 9907 patients	Increased mortality strongest in F→M	No clear harm	HR ~1.08 per mismatched unit
Desmarets 2016 [94]	Cardiac surgery, FR, 2715 patients	No significant effect (adjusted HR ~0.96)	Trend toward increased mortality (HR ~2.0, not significant)	HR ~2.28 unadjusted; effect disappeared after adjustment
Zeller 2019 [93]	5 observational studies, 86,737 patients	Pooled increased mortality (HR ~1.13)	Not separated	Reported as “sex-mismatched”
Alshalani 2021 [98]	Mechanistic, ICU, 69 patients	Higher syndecan-1 and sTM → endothelial activation	Included, but effect driven by mismatched group	Sex-mismatched group showed higher injury markers
Alshalani 2022 [96]	ICU, 6992 patients (403 unisex transfused)	Increased ICU mortality in F→M vs. F→F (OR 2.43)	No significant difference	Mismatched group: trend to ↑ ARDS, ↓ AKI
Li 2025 [97]	ICU, retrospective, ~500 patients	No mortality/sepsis effect	In women, M→F units gave larger Hb increments	No sepsis difference by donor sex
Bruun-Rasmussen 2022 [99]	Registry-based, causal inference analysis, SE, hundreds of thousands recipients	Not separated	Not separated	Sex-matched transfusions associated with modest survival benefit
Chassé 2016 [90]	Observational, CA, 30,503 recipients	Increased mortality with female donor blood, especially in male recipients	Increased risk also seen in female recipients	Effect across donor sex (HR 1.08 per female donor unit)
Edgren 2017 [91]	Observational, SE/DK, 968,264 recipients	No association after adjustment	No association	Null in fully adjusted models
Zhao 2022 [50]	Natural experiment, SE, 368,778 recipients	No survival difference (−0.1%)	No survival difference	No effect by donor sex or parity
Chassé 2023 [19]	RCT, CA, 8719 patients randomized	No survival difference	No survival difference	No overall effect (HR 0.98)

Abbreviations: F→M, male recipient of female donor blood; M→F, female recipient of male donor blood; HR, hazard ratio; OR, odds ratio; NS, not significant; Hb, hemoglobin; ICU, intensive care unit; ARDS, acute respiratory distress syndrome; AKI, acute kidney injury; sTM, soluble thrombomodulin; RCT, randomized controlled trial; NL, Netherlands; SE, Sweden; FR, France; CA, Canada; DK, Denmark. Strength of evidence: Observational cohorts = low to moderate certainty (risk of residual confounding); Meta-analysis of observational studies = very low certainty; Mechanistic ICU studies = exploratory/biological plausibility only; Randomized controlled trial = high certainty; ↑: increase; ↓: decrease.

## Data Availability

No new data were created or analyzed in this study. Data sharing is not applicable to this article.

## Abbreviations

The following abbreviations are used in this manuscript:

AABB	Association for the Advancement of Blood & Biotherapies
AKI	Acute Kidney Injury
ARDS	Acute Respiratory Distress Syndrome
CBC	Complete Blood Count
CI	Confidence Interval
CPD	Citrate–Phosphate–Dextrose
CPDA-1	Citrate–Phosphate–Dextrose–Adenine
DIC	Disseminated Intravascular Coagulation
FFP	Fresh Frozen Plasma
F→M	Female donor to Male recipient (transfusion)
GLRX	Glutaredoxin-1
GPX4	Glutathione Peroxidase 4
G3P	Glyceraldehyde-3-Phosphate
G6PD	Glucose-6-Phosphate Dehydrogenase
Hct	Hematocrit
Hb	Hemoglobin
HLA	Human Leukocyte Antigen
HNA	Human Neutrophil Antigen
HR	Hazard Ratio
ICU	Intensive Care Unit
MCV	Mean Corpuscular Volume
MCHC	Mean Corpuscular Hemoglobin Concentration
M→F	Male donor to Female recipient (transfusion)
NHSBT	National Health Service Blood and Transplant (UK)
OR	Odds Ratio
PAS	Platelet Additive Solution
PLTs	Platelets
PRP	Platelet-Rich Plasma
PTP	Post-Transfusion Purpura
QC	Quality Control
RBCs	Red Blood Cells
RCT	Randomized Controlled Trial
ROS	Reactive Oxygen Species
sTM	Soluble Thrombomodulin
SHOT	Serious Hazards of Transfusion (UK haemovigilance)
TRALI	Transfusion-Related Acute Lung Injury
TRIM	Transfusion-Related Immunomodulation
vWD	von Willebrand Disease
WB	Whole Blood
WHO	World Health Organization
